# Serial extracellular volume quantification using cardiac magnetic resonance imaging in transthyretin amyloidosis patients treated with tafamidis

**DOI:** 10.1007/s00330-025-11792-x

**Published:** 2025-07-16

**Authors:** Franz Duca, Michael Poledniczek, Christina Kronberger, Christina Binder, René Rettl, Luciana Camuz-Ligios, Hermine Agis, Matthias Koschutnik, Carolina Donà, Roza Badr-Eslam, Dietrich Beitzke, Christian Loewe, Christian Nitsche, Christian Hengstenberg, Johannes Kastner, Jutta Bergler-Klein, Andreas Anselm Kammerlander

**Affiliations:** 1 Division of Cardiology, Department of Internal Medicine II, Medical University of Vienna, Vienna, Austria; 2 Division of Hematology, Department of Internal Medicine I, Medical University of Vienna, Vienna, Austria; 3 Division of Cardiovascular and Interventional Radiology, Department of Bioimaging and Image-Guided Therapy, Medical University of Vienna, Vienna, Austria

**Keywords:** Transthyretin amyloidosis, Tafamidis, Cardiac MRI, ECV, Outcome

## Abstract

**Objectives:**

Cardiac transthyretin amyloidosis (ATTR CA) has been increasingly recognized as an important heart failure (HF) entity, and cardiac magnetic resonance (CMR) imaging is a mainstay in the clinical evaluation of this disease. However, studies evaluating the prognostic values of longitudinal data in ATTR CA patients with disease-modifying therapies are lacking. We aimed to assess the prognostic significance of serial quantification of extracellular volume (ECV) in ATTR CA patients treated with tafamidis.

**Materials and methods:**

The present study included ATTR CA patients who received ≥ 3 months of tafamidis treatment and underwent baseline and CMR, including ECV quantification. The primary endpoint was a composite of all-cause mortality, cardiac transplantation, or hospitalization due to HF.

**Results:**

Between June 2016 and June 2020, 54 patients were included in the present analysis and were representative of a typical ATTR CA cohort (median age: 76.7 years, male participants: 79.6%). The median time on tafamidis before follow-up CMR was 6.0 months (interquartile range (IQR): 6.0–8.3). Participants depicted typical structural changes for ATTR CA patients with markedly elevated ECV (51.4% (IQR: 41.3–57.6), normal range: 20–32%) and myocardial ventricular hypertrophy (intraventricular septum: 19 mm (16.0–22.0), normal range: 5–12 mm). Change of ECV was the only parameter among clinical, laboratory, and CMR parameters that was independently associated with the composite endpoint (HR: 1.077, 95% CI: 1.013–1.145, *p* = 0.017).

**Conclusions:**

Change of ECV was the only predictor of adverse outcome among clinical, laboratory, and imaging parameters in our cohort of tafamidis-treated ATTR CA patients.

**Key Points:**

***Question*** *What is the prognostic significance of serial ECV quantification in transthyretin amyloidosis patients on tafamidis treatment*?

***Findings*** *Change of ECV is a strong predictor of a combined endpoint consisting of all-cause mortality, cardiac transplantation, or hospitalization due to HF*.

***Clinical relevance*** *Patients with increasing ECV might potentially benefit from a change in amyloid-specific treatment to a different agent*.

**Graphical Abstract:**

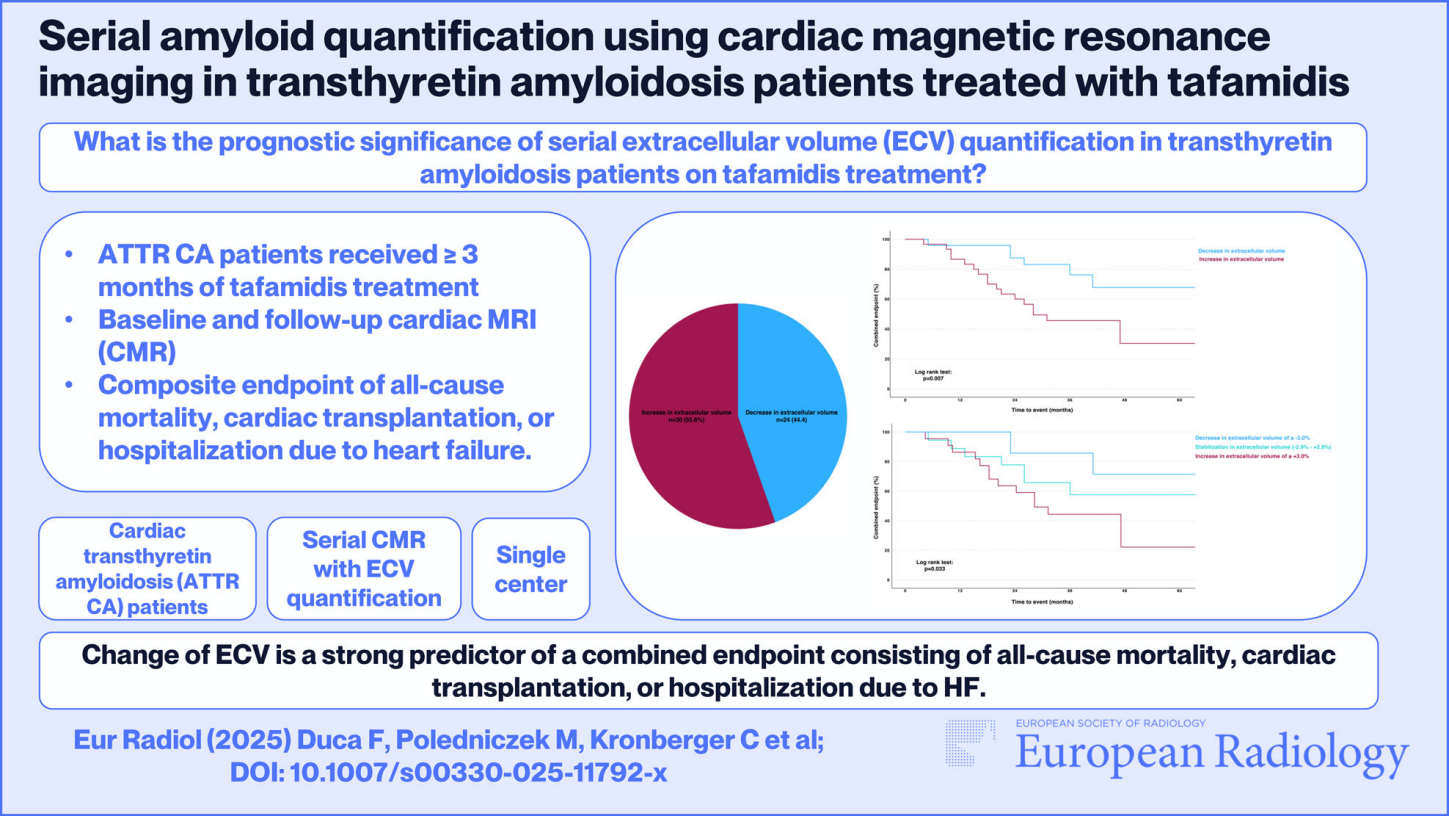

## Introduction

Over the past decade, cardiac transthyretin amyloidosis (ATTR CA) has gained recognition as a significant cause of heart failure (HF), with affected patients facing a poor prognosis, particularly in advanced stages of the disease [1]. The pathophysiological hallmark of CA is the deposition of misfolded proteins (amyloid) within the myocardial extracellular space, leading to an expansion of the extracellular volume (ECV) [2, 3]. The gold standard for ECV quantification is histological assessment of amyloid burden in endomyocardial biopsy (EMB). However, due to the procedural risks and the heterogeneous distribution of amyloid in the myocardium, EMB is not suitable for serial ECV assessment. Cardiac magnetic resonance (CMR) T1-mapping has emerged as a validated, non-invasive method for ECV quantification in various cardiac conditions, including CA [4, 5].

Rettl et al could demonstrate that treatment of ATTR CA patients with the TTR stabilizer tafamidis has beneficial effects on disease progression, as suggested by improvements in CMR and 99mTc-DPD SPECT/CT examinations [6, 7]. Patients receiving Tafamidis treatment showed a stabilization with respect to ECV (47.5% vs 47.7%), while treatment-naïve patients increased (49.3% vs 54.6%). With respect to nuclear imaging parameters, Tafamidis led to a reduction in SUV retention index from 5.96 g/mL to 3.27 g/mL.

Given that ATTR cardiac amyloidosis (CA) has become a treatable condition and with several new agents on the horizon, it is crucial to develop methods for identifying patients who will benefit most from specific treatments, particularly considering the substantial costs associated with these therapies [8–12]. In a recent study by our group, it was demonstrated that the change of amyloid burden is a strong predictor of outcome in a cohort of CA patients [13]. However, dedicated studies have yet to be performed investigating the prognostic relevance of serial ECV quantification in tafamidis-treated ATTR CA patients.

We hypothesize that ATTR CA patients treated with tafamidis who exhibit a decreasing amyloid burden, assessed through serial CMR T1-mapping, may have better outcomes, potentially identifying those with the greatest clinical benefit.

## Material and methods

### Setting and study design

This retrospective analysis consisted of patients from a CA registry enrolled between June 2016 and June 2020. Patients were referred from within our institution, external outpatient clinics, or other hospitals. The study was approved by the ethics committee of the Medical University of Vienna (EK# 796/2010), and written informed consent was obtained from all participants prior to enrollment. The study was conducted at the Department of Cardiology, Medical University of Vienna, which features a dedicated amyloidosis outpatient clinic and a multimodality imaging laboratory.

### Diagnosis of ATTR CA

ATTR CA was diagnosed when patients exhibited Perugini grade ≥ 2 myocardial tracer uptake on bone scintigraphy, and the presence of a paraprotein was ruled out [14]. For patients with inconclusive non-invasive test results, ATTR amyloidosis was confirmed through EMB samples that stained positive with Congo red, exhibited apple-green birefringence under polarized light, and reacted with anti-ATTR antibodies. Gene sequencing was offered to all patients diagnosed with ATTR CA.

### National Amyloidosis Center (NAC) staging system

Disease severity was assessed using the expanded NAC staging system, which stratifies ATTR CA patients according to NT-proBNP and estimated glomerular filtration rate (eGFR) levels. Stage 1 is defined as N-terminal N-terminal pro-brain natriuretic peptide (NT-proBNP) ≤ 3000 pg/mL and eGFR ≥ 45 mL/min/1.73 m^2^, stage 2 is defined as either NT-proBNP > 3000 pg/mL or eGFR < 45 mL/min/1.73 m^2^, stage 3 is defined as NT-proBNP > 3000 pg/mL and eGFR < 45 mL/min/1.73 m^2^, and stage 4 is defined as NT-proBNP ≥ 10,000 pg/mL [15].

**Fig. 1:**
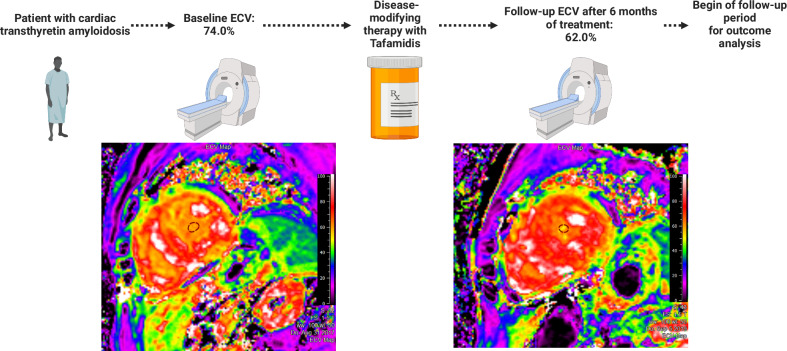
Study timeline and ECV maps for a representative patient. Patients underwent baseline cardiac magnetic resonance (CMR) imaging with ECV quantification. Follow-up CMR was performed after a median treatment with tafamidis for 6 months and also marked the beginning of the follow-up period for outcome analysis. ECV at baseline for the depicted patient was 74.0%. He then received tafamidis for 6 months before follow-up ECV quantification, which decreased to 62.0%. The patient was then followed for 43 months and did not reach the endpoint of all-cause mortality, cardiac transplantation, or hospitalization due to HF

### Study schedule (Fig. 1)

Baseline and follow-up visits of study participants consisted of CMR imaging with T1 mapping. Clinical and laboratory assessment included New York Heart Association (NYHA) class, 6-min walk distance (6-MWD), NT-proBNP, troponin T, eGFR, and NAC stage. As per the study registry protocol, follow-up was scheduled between 6 and 24 months after baseline investigation. However, patients were not excluded from the present analysis if their follow-up was out of the scheduled range, if they received ≥ 3 months of Tafamidis treatment, and underwent baseline, as well as follow-up CMR.

### Cardiac magnetic resonance imaging (CMR)

CMR indications for baseline and follow-up CMR were either due to clinical indication/or research purposes within the context of our CA patient registry. Neither acquisition protocols nor the scanner changed throughout the course of the study.

All cardiovascular MRI studies, including late gadolinium enhancement imaging, were performed on a 1.5-T system and included cine imaging, as well as late gadolinium enhancement images (LGE), which were acquired at least 10 min after injection of 0.1 mmol/L gadobutrol.

T1 mapping was performed with electrocardiographically triggered MOLLI and ECV calculation was performed as previously described [16–18]. Regions of interest for ECV quantification were drawn in a mid-cavity short axis and a 4-chamber view by a single reader (FD) who is a board-certified cardiologist with > 10 years of experience in CMR imaging. In all patients, venous blood for conventional hematocrit measurement was drawn when placing the intravenous line for contrast agent administration. ECV was calculated by using the previously described formula (Δ indicates the change from native and postcontrast mapping results, T1_myocardium_ is T1 times of the LV, and T1_blood_ is the blood pool) [18]:

$${{\rm{ECV}}}=(1-{{\rm{hematocrit}}})* (\Delta [1/{{\rm{T}}}1{{\rm{myocardium}}}]/\Delta [1/{{\rm{T}}}1{{\rm{blood}}}])$$

### Outcome measures

Due to the study cohort size, the primary outcome measure was a composite of all-cause mortality, cardiac transplantation, or hospitalization due to HF.

### Statistical analysis

Continuous variables are expressed as median and interquartile range (IQR). Categorical variables are presented as numbers and percentages. Continuous variables were compared using the Wilcoxon signed-rank for paired and the Mann–Whitney *U*-test for non-paired variables. McNemar’s test was used for paired categorical parameters. Kaplan–Meier plots and respective log-rank tests were used to verify the time-dependent discriminative power of the parameters of interest. Cox regression models and Kaplan–Meier curves were calculated to assess the effect of CMR parameters on event-free survival. For our multivariable Cox regression model, we adjusted for NAC stage at baseline [1]. To avoid immortal time bias in our outcome analysis, the date of follow-up CMR was used as the starting point for the follow-up period in Kaplan–Meier curves and Cox regression models when evaluating the predictive value of follow-up parameters and changes (Δ) in these parameters. Events occurring before the follow-up CMR were not included in these analyses. IBM SPSS version 27.0 and STAT 16 were used for statistical analysis. The graphical abstract was created with Biorender.com. A *p* value of ≤ 0.05 was set as the level of significance.

## Results

### Patient population and study cohort

Between June 2016 and June 2020, 54 patients from our registry with ATTR CA who received ≥ 3 months of treatment with tafamidis and underwent baseline, as well as follow-up CMR with ECV quantification, were included in the present analysis. Patients who were included before 2019 received 20 mg once daily (*n* = 16), while the remaining patients in the present study received 61 mg once daily. A total of 78 patients were not included in the analysis because they had other forms of CA. Further 96 patients were excluded because they either did not undergo baseline CMR (pacemaker: 23, patient decision: 8, implantable cardioverter-defibrillator: 6, unknown reason: 5, eGFR < 30 mL/min/1.73 m^2^: 3, claustrophobia: 2, other non-CMR compatible implant: 1 or follow-up CMR (CMR pending: 15, eGFR < 30 mL/min/1.73 m^2^: 8, death before follow-up CMR: 7, unknown reason: 5, pacemaker: 5, patient decision: 4, implantable cardioverter-defibrillator: 3, claustrophobia: 1). 23 patients did not receive any amyloid-specific therapies, while 2 were on gene silencers and were therefore not included in the present study. These 23 patients represent a historical cohort who underwent baseline and follow-up CMR before Tafamidis became commercially available in Austria.

33 patients from our registry who received Tafamidis treatment were excluded from our analysis due to missing follow-up CMR (follow-up CMR pending: *n* = 19, eGFR < 30 mL/min/1.73 m^2^: *n* = 5, pacemaker: *n* = 4, implantable cardioverter defibrillator: *n* = 3, patient decision: *n* = 2).

The final cohort consisted of 48 (88.9%) patients with wild-type ATTR (ATTRwt) and 6 (11.1%) with variant ATTR (ATTRv) CM (Val40Ile: *n* = 1, Thr80Arg: *n* = 1, His80Arg: *n* = 4). Median treatment time on tafamidis before follow-up CMR was 6.0 months (IQR: 6.0–8.3) while the median time difference between baseline and follow-up CMR was 10.0 months (IQR: 8.0–14.3). Median follow-up time in our study was 31.0 months (IQR: 23.0–41.0).

### Baseline and follow-up characteristics of the tafamidis-treated cohort

Baseline and follow-up characteristics of our study cohort are depicted in Table 1. The median changes in clinical and CMR parameters are shown in Table 2.

**Table 1:** Baseline and follow-up parameters of the tafamidis-treated cohort

Variable	Baseline (*n* = 54)	Follow-up (*n* = 54)	*p* value
Clinical parameters			
Age, years	76.7 (70.9–81.8)	77.9 (71.6–82.6)	**<** **0.001**
Sex, male gender, *n*	43 (79.6)	43 (79.6)	n.a
NYHA functional class ≥ III, *n*	18 (33.3)	10 (18.5)	0.077
6-min walk test, m (*n* = 45)	442 (329–502)	426 (348–512)	0.957
NT-proBNP, pg/mL	1674 (702–3225)	2262 (1015–2873)	0.493
Troponin t, ng/L (*n* = 14)	43.0 (29.0–64.0)	51.5 (30.5–71.8)	**0.021**
eGFR, mL/min/1.73 m^2^	59.5 (46.0–80.7)	54.9 (43.2–68.0)	**0.030**
eGFR < 45 mL/min/1.73 m^2^	12 (22.2)	16 (29.6)	0.344
NT-proBNP > 3000 pg/mL	14 (25.9)	12 (22.2)	0.754
NAC stage 1	35 (64.8)	31 (57.4)	0.424
NAC stage 2	10 (18.5)	18 (33.3)	0.687
NAC stage 3	7 (13.0)	5 (9.3)	0.118
NAC stage 4	1 (1.9)	0 (0.0)	1.000
CMR parameters			
Myocardial native T1 time, ms	1102 (1075–1147)	1108 (1080–1145)	0.39
ECV, %	51.4 (41.3–57.6)	50.9 (43.9–60.3)	0.11
Interventricular septum, mm	19.0 (16.0–22.0)	19.0 (16.7–23.4)	0.86
Left ventricular mass, g	197 (159–236)	195 (151–235)	0.77
Left atrial area, cm^2^	32.0 (27.8–39.0)	32.5 (29.0–37.5)	0.79
Right atrial area, cm^2^	31.0 (26.0–37.0)	30.7 (26.6–37.2)	0.83
Left ventricular global longitudinal strain, %	−1.9 (−14.6 to 9.8)	−12.8 (−14.5 to −9.5)	0.86
Left ventricular ejection fraction, %	55.5 (46.5–61.1)	50.3 (42.0–59.0)	**0.02**
Left ventricular cardiac output, L/min	5.6 (4.8–6.3)	5.6 (4.4–6.6)	0.49
Left ventricular end-diastolic volume, mL	159 (146–205)	169 (151–207)	**0.02**
Right ventricular ejection fraction, %	49.0 (40.8–55.3)	45.3 (36.8–50.7)	**0.00**
Right ventricular cardiac output, L/min	5.3 (4.2–6.1)	5.1 (4.1–6.2)	0.32
Right ventricular end-diastolic volume, mL	177 (141–217)	176 (150–221)	**0.02**
Pulmonary artery, mm	28.0 (25–31.0)	28.0 (25.0–30.6)	0.71
Pleural effusion, *n*	10 (18.5)	19 (35.2)	**0.01**
Pericardial effusion, *n*	18 (33.3)	18 (33.3)	1.00

Numbers in brackets are % for dichotomous and IQR for continuous variables

Bold indicates statistical significance (*p* < 0.05)

*NYHA* New York Heart Association, * eGFR* estimated glomerular filtration rate, * n.a* not applicable

**Table 2:** Median changes of clinical and CMR parameters

Variable		*p* value
Clinical parameters		
Δ in 6-min walk test, m	0.0 (−43.5 to 46.5)	0.957
Δ in NT-proBNP, pg/mL	84.3 (−515 to 764)	0.493
Δ in Troponin t, ng/L (*n* = 14)	−1.0 (−18.3 to 1.0)	**0.021**
Δ in eGFR, mL/min/1.73 m^2^	−2.6 (−15.2 to 3.8)	**0.030**
CMR parameters		
Δ in Myocardial native T1 time, ms	8.0 (−20.0 to 26.5)	0.39
Δ in ECV, %	2.2 (−3.2 to 5.5)	0.11
Δ in Interventricular septum, mm	1.0 (−1.3 to 3.2)	0.86
Δ in Left ventricular mass, g	−0.1 (−20.3 to 19.8)	0.77
Δ in Left atrial area, cm^2^	−0.1 (−3.0 to 3.3)	0.79
Δ in Right atrial area, cm^2^	0.0 (2.4–3.0)	0.83
Δ in Left ventricular global longitudinal strain, %	−0.5 (−1.4 to 2.3)	0.86
Δ in Left ventricular ejection fraction, %	−3.0 (−9.8 to 1.6)	**0.02**
Δ in Left ventricular cardiac output, L/min	0.03 (−0.7 to 0.9)	0.49
Δ in Left ventricular end-diastolic volume, mL	5.0 (−4.8 to 24.3)	**0.02**
Δ in Right ventricular ejection fraction, %	−3.8 (−9.7 to 2.0)	**0.00**
Δ in Right ventricular cardiac output, L/min	−0.2 (−1.0 to 0.8)	0.32
Δ in Right ventricular end-diastolic volume, mL	8.0 (−14.0 to 32.5)	**0.02**
Δ in Pulmonary artery diameter, mm	0.0 (−2.9 to 2.0)	0.71

Numbers in brackets are the IQR for continuous variables

*p* value indicates the difference between median baseline and follow-up values

Bold indicates statistical significance (*p* < 0.05)

*eGFR* estimated glomerular filtration rate

Our study cohort consisted mostly of elderly (76.7 years, (IQR: 70.9–81.8)) men (*n* = 43, 79.6%). One third was in NYHA class ≥ 3, median 6-min walking test distance was 442 m (IQR: 329–502), and NT-proBNP, as well as troponin T, were elevated with 1674 pg/mL (IQR: 702–3225) and 43 ng/L (IQR: 29.0–64.0). When patients were characterized with the NAC staging system, 64.8% were in stage 1, 18.5% in stage 2, 13.0% in stage 3, and 1.9% in stage 4.

With respect to myocardial structural characterization patients had markedly elevated ECV (51.4% (IQR: 41.3–57.6)) accompanied by increased left ventricular (LV) mass (197 g (IQR: 159–236)) and hypertrophy (intraventricular wall thickness (IVS): 19.0 mm (IQR: 16.0–22.0)).

**Fig. 2:**
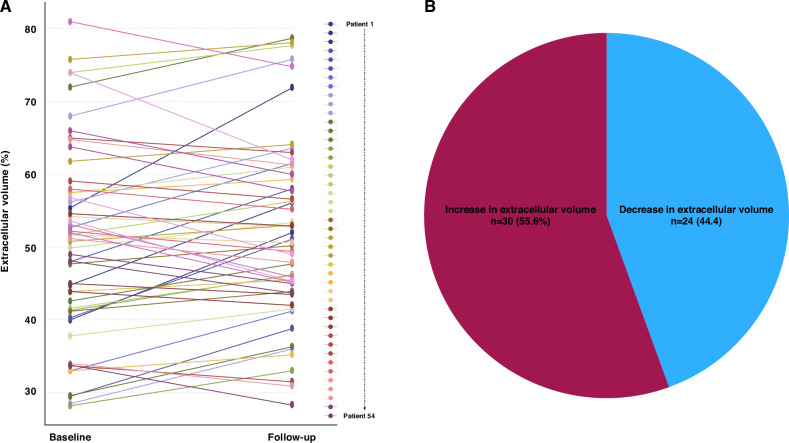
ECV at baseline and follow-up for individual patients, and a Pie chart for patients with increasing and decreasing ECV. **A** At follow-up, a total of 30 patients (55.6%) increased with their ECV amount, while the remaining 24 (44.4%) had decreasing ECV. **B** At follow-up, a total of 30 patients (55.6%) increased with their ECV amount, while the remaining 24 (44.4%) had decreasing ECV

While NT-proBNP values did not change significantly (*p* = 0.493) from baseline to follow-up, Troponin T increased from 43.0 ng/L to 51.5 ng/L (*p* = 0.021) and eGFR decreased from 59.5 mL/min/1.73m2 to 54.9 mL/min/1.73m2 (*p* = 0.030). Structurally, patients did neither display significant changes in mean ECV (*p* = 0.107), nor in LV mass (*p* = 0.768), nor in IVS (*p* = 0.863.). Individual changes of ECV from baseline to follow-up are depicted in Fig. 2A, and stratification of ECV changes (increase vs decrease) is shown in Fig. 2B, respectively. Quantification of ECV showed excellent intra-observer agreement with an intraclass correlation coefficient of 0.97.

However, we could detect deteriorations in LV and right ventricular ejection fraction (LVEF, RVEF) from 55.5% to 50.3% (*p* = 0.018) and 49.0% to 45.3% (*p* = 0.004), respectively.

### Predictors of outcome in tafamidis-treated patients

**Fig. 3:**
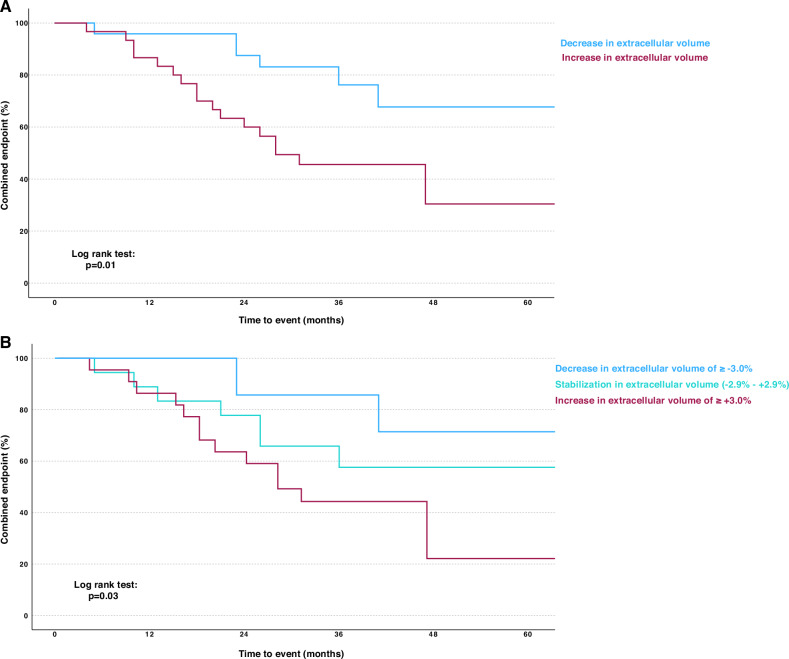
Kaplan–Meier curves stratified according to change in ECV. Change of ECV from baseline to follow-up was predictive of the combined endpoint of all-cause mortality, cardiac transplantation, or hospitalization due to HF. **A** Panel depicts patients dichotomized according to increasing vs decreasing in ECV (log rank test: *p* = 0.01) while (**B**) shows patients stratified into cohorts with decreasing (≥ −3.0%), stable (−2.9% to +2.9%), and increasing ECV (≥ +3.0%) (log rank test: *p* = 0.03)

When we assessed the prognostic significance of changes from baseline to follow-up of clinical, laboratory, and CMR parameters, we found that only Δ ECV was independently associated with the composite endpoint of all-cause mortality, cardiac transplantation, or hospitalization due to HF (HR: 1.077, 95% CI: 1.013–1.145, *p* = 0.02). When Δ ECV was analyzed as a dichotomized variable (increase vs decrease), it remained associated with the composite endpoint, even after adjusting for baseline NAC stage (HR: 3.523, 95% CI: 1.388–8.938, *p* = 0.01) (Table 3). Respective Kaplan–Meier curves diverged after approximately 9 months. They continued to diverge throughout the remainder of the follow-up period [Fig. 3A (*p* = 0.01)]. In a second analysis, patients were stratified according to decreasing (≥ −3.0%), stable (−2.9% to +2.9%), and increasing (≥ +3.0%) ECV. In line with our first Kaplan-Meier analysis, patients with an increase of ECV ≥ +3.0% had the worst, while patients with a decrease of ≥ −3.0% had the best survival (Fig. 3B (*p* = 0.03)). Of note, the third cohort, which consisted of patients with stable ECV, added more granularity to our outcome data. Baseline ECV did not show a statistically significant association with the composite endpoint in our cohort (HR: 1.004, 95% CI: 0.971–1.037, *p* = 0.83).

**Table 3:** Cox regression analyses for the composite endpoint of all-cause death, cardiac transplantation, or HF hospitalization

	Univariable regression		Multivariable regression			
Variable	Crude hazard ratio	95% Confidence interval	*p* value	Adjusted hazard ratio	95% Confidence interval	*p* value
Clinical parameters						
Δ in six-min walk test distance	1.006	1.000–1.012	0.055	1.006	1.000–1.012	0.055
Laboratory parameters						
Δ in NT-proBNP, per quartile	1.023	0.713–1.467	0.903	0.686	0.359–1.311	0.254
Δ in troponin T, per ng/L	1.008	0.982–1.036	0.540	1.009	0.982–1.036	0.507
Δ in eGFR, per mL/min/1.73 m^2^	1.014	0.987–1.041	0.319	1.016	0.988–1.045	0.267
CMR parameters						
Δ in Myocardial native T1 time, ms	1.005	0.994–1.017	0.37	1.005	0.993–1.017	0.44
Δ in ECV, %	1.075	1.009–1.146	**0.03**	1.077	1.013–1.145	**0.02**
ECV at baseline, %	1.004	0.971–1.037	0.83	1.002	0.970–1.034	0.27
Increase in ECV at follow-up	3.328	1.314–8.433	**0.01**	3.523	1.388–8.938	**0.01**
Δ in Interventricular septum, mm	1.019	0.957–1.085	0.56	1.013	0.951–1.079	0.68
Δ in Left ventricular mass, g	1.000	0.991–1.009	1.00	1.002	0.992–1.013	0.70
Δ in Left atrial area, cm^2^	1.001	0.941–1.066	0.97	1.018	0.949–1.093	0.61
Δ in Right atrial area, cm^2^	0.997	0.948–1.047	0.90	1.006	0.956–1.060	0.81
Δ in Left ventricular global longitudinal strain, %	0.961	0.815–1.132	0.63	0.979	0.823–1.164	0.80
Δ in Left ventricular ejection fraction, %	0.986	0.950–1.023	0.46	0.986	0.951–1.022	0.44
Δ in Left ventricular cardiac output, L/min	1.325	0.962–1.825	0.09	1.306	0.956–1.783	0.09
Δ in Left ventricular end-diastolic volume, mL	0.991	0.977–1.044	0.18	0.994	0.979–1.009	0.44
Δ in Right ventricular ejection fraction, %	1.024	0.981–1.069	0.28	1.018	0.974–1.065	0.43
Δ in Right ventricular cardiac output, L/min	0.946	0.694–1.289	0.72	0.953	0.704–1.289	0.75
Δ in Right ventricular end-diastolic volume, mL	0.989	0.978–1.000	0.05	0.991	0.980–1.003	0.13
Δ in Pulmonary artery diameter, mm	0.900	0.778–1.041	0.16	0.920	0.794–1.066	0.27

Multivariable model adjusted for baseline NAC stage

T0 was the date of follow-up CMR

Bold indicates statistical significance (*p* < 0.05)

To minimize the potential influence of the time range between baseline and follow-up CMR, we computed an additional Cox-regression analysis excluding patients with < 6 (*n* = 2) and > 24 months (*n* = 6). In this model ECV (per 1% increase) and ECV (increase vs decrease) remained predictive of the composite endpoint, even after adjustment for NAC staging with a HR of 1.070 (95% CI: 1.004–1.141, *p* = 0.04), and a HR of 3.476 (95% CI: 1.313–9.204, *p* = 0.01), respectively.

## Discussion

In our study of 54 ATTR CA patients who received treatment with the TTR stabilizer tafamidis and underwent baseline, as well as follow-up CMR with T1 mapping, we could detect that an increase in ECV was associated with the composite endpoint of all-cause mortality, cardiac transplantation, or hospitalization due to HF.

### Predictors of outcome in transthyretin CA

In recent years, several studies investigating predictors of outcome in large cohorts could advance the field of risk-stratification in ATTR CA significantly [15, 19–22]. However, the aforementioned trials involved heterogeneous patient cohorts regarding treatment status. Some patients received amyloidosis-modifying treatments, others participated in placebo-controlled drug trials, and some were entirely treatment-naïve. With tafamidis now available in over 55 countries, including the United States of America, the United Kingdom, Australia, Brazil, and most of Europe, the proportion of ATTR CA patients not receiving disease-modifying treatment is becoming a minority. In 2024, two positive phase III trials, ATTRibute-CM, evaluating Acoramidis, and Helios-B, evaluating Vutrisiran, were published, and regulatory approval from the EMA and FDA for both drugs is anticipated in 2025 [8, 9]. Thus, a clinical need for refined risk stratification in tafamidis-treated patients arises, as clinicians seek to identify those who might benefit from adjustments in therapy.

In our cohort of Tafamdis-treated ATTR CA patients, the change of ECV between baseline and follow-up CMR was the sole parameter, among clinical, laboratory, and CMR metrics associated with adverse outcomes. However, several factors might have contributed to the lack of statistical significance among other potential predictors of outcome in our study population. A major factor might be that our cohort consisted exclusively of Tafamidis-treated ATTR-CA patients [23]. Furthermore, outcome events between baseline and follow-up assessment had to be excluded to avoid an immortal time bias. Also, considering that treated ATTR-CA patients nowadays often have very good long-term outcomes, our relatively short follow-up period of 31.0 months may have influenced the outcome analyses [24].

Given the facts that ECV reflects histologically quantified myocardial amyloid burden, and amyloid deposition within the extracellular matrix of the myocardium is the pathophysiological hallmark feature of this disease, changes in ECV may indicate disease in treated regression and also potentially reflect successful treatment response [3, 4]. However, ECV is not solely a measure of amyloid burden; it is also influenced by myocardial fibrosis and edema, such as collagen accumulation [17, 25, 26]. Both fibrosis and edema are present in CA, with edema potentially reflecting amyloid toxicity to the myocardium [27, 28]. Consequently, a reduction in ECV might not only represent a decrease in amyloid burden but also a reduction in myocardial edema in our cohort. Nonetheless, this hypothesis requires histological validation.

### Limitations

A major limitation of the present study is its relatively small sample size and potential selection bias, as 199 (78 non-ATTR CA, 96 either no baseline or follow-up CMR, 25 did not receive Tafamidis) patients from our registry had to be excluded from the present analysis. Nevertheless, our cohort still adds a significant number of Tafamidis-treated ATTR CA patients to the literature. Additionally, the single-center design of our study may have introduced a center-specific bias; nevertheless, limiting data collection to one center provides benefits such as consistent quality of assessment, adherence to a uniform clinical routine, and standardized follow-up. Furthermore, the relatively short median follow-up period (31.0 months) and time on tafamidis treatment (6.0 months) in our cohort call for studies with longer treatment and follow-up periods. Yet, data from the ATTRACT trial, which demonstrated treatment effects on clinical parameters such as the 6-min walk distance and quality of life after 6 months, suggest that time-on-treatment in our cohort was long enough to detect meaningful changes. Furthermore, the fact that only patients who underwent CMR were included in this study limits the generalizability of our data to cohorts with implanted cardiac devices or other contraindications to CMR. A further limitation of our study is the inconsistent time intervals between baseline and follow-up CMR.

## Conclusions

The present study assessed the change of ECV as a surrogate for amyloid burden, assessed by serial CMR T1 mapping. It was the only predictor of adverse outcomes among clinical, laboratory, and imaging parameters in tafamidis-treated ATTR CA patients. Thus, serial ECV measurements might aid in guiding future treatment decisions (e.g., which patients should switch from TTR stabilizers to TTR gene silencers or depleters) in this difficult-to-treat patient population. However, larger prospective external validation studies are needed to confirm our findings.

## Abbreviations

ATTR CA: Cardiac transthyretin amyloidosis
CA: Cardiac amyloidosis
CMR: Cardiac magnetic resonance imaging
ECV: Extracellular volume
eGFR: Estimated glomerular filtration rate
EMB: Endomyocardial biopsy
HF: Heart failure
NAC: National Amyloidosis Center
NT-proBNP: N-terminal pro-brain natriuretic peptide
NYHA: New York Heart Association
